# Simulation-Based Education for Enhancing Obstetric Emergency Response: A Needs Impact Evaluation

**DOI:** 10.7759/cureus.43908

**Published:** 2023-08-22

**Authors:** Aderemi O Alalade, Sindhu Sekar

**Affiliations:** 1 Obstetrics and Gynaecology, Wrexham Maelor Hospital, Wrexham, GBR

**Keywords:** medical education, training, obstetrics emergencies, evaluation, simulation

## Abstract

Simulation is an ideal method for procedural training in obstetrics. To maximise training opportunities through simulation, the evaluation of these educational activities should be based on a standardised evidence-based approach. As such, the tools used in the evaluative process should be validated for content and context, as this ensures consistency of approach. It also makes the findings and recommendations acceptable, applicable and credible. More so, the information can be used for planning further learning, assessment of the competency of the trainers and educational governance purposes. In our view, simulation should be used in conjunction with other forms of procedural assessment such as mini-clinical examinations and case-based discussions to translate skills to actual life events. The learners will be able to further consolidate their learning, improve professional skills and feel involved throughout the programme.

## Introduction and background

Obstetrics is a discipline where unexpected emergencies occur, which could be fatal and possibly lead to severe maternal morbidity and mortality. The labour ward has the function of catering to complicated and uncomplicated maternal deliveries and being ready for intervention in cases of obstetric emergencies [1]. The management of these emergencies is often a collaborative effort involving multiple disciplines such as obstetricians, midwives, anaesthetists, operating room assistants, healthcare assistants and paediatricians. In many of these instances, these clinical encounters require high acuity: if the staff is unprepared, the outcomes could be catastrophic. Clinicians managing these cases require complex practical (technical) and soft (non-technical) skills that may be difficult to teach through didactic lectures or in clinical settings [1].

Obstetric simulation is the recreation of potential emergencies in the labour ward with real-life barriers. The primary objective is to provide learners with the opportunity to acquire the necessary skills to maintain control over these situations and minimise harm to patients [2]. Also, to maximise its educational benefits, these learners are expected to react to these scenarios as they would in real life. Simulation of obstetric emergencies (SOE) has been shown to improve knowledge, teamwork abilities, leadership skills, and decision-making in acute obstetric events [3,4]. However, to maximise the benefits of SOE, it is necessary to continuously evaluate local programmes for barriers to their delivery and opportunities to improve their educational content to sustain or improve clinical outcomes [5]. Several parameters can be evaluated in SOE, including knowledge, teamwork, communication, situation awareness and cost-effectiveness. This paper describes a framework for the effective delivery of SOE, effective quality assurance processes and how to assess the impact of the service on clinical outcomes.

Effective delivery of SOE

Simulation, as an assessment for learning (formative assessment), is useful to define and demonstrate the progression of a learner's performance at technical skills, cognitive tasks, and their psychomotor and affective abilities [6]. It is also a useful tool to elicit non-technical skills such as teamworking abilities, decision-making and empathy [6]. It draws its relevance from the principles that characterise how adults learn. Simulation is experiential learning but multi-theorised and includes aspects of cognitive, humanistic, behavioural, and social and transformational learning theories, to mention a few [7]. Adult learning is largely self-motivational, which promotes self-directed learning and helps the learner to optimise learning opportunities [8]. Adults learn through a process of dissonance, elaboration, refinement, organisation, feedback and then consolidation of new knowledge [7]. SOE must integrate these learning processes through a pre-brief, meaning interactions during the simulation and a debrief afterwards (feedback).

 *Pre-brief*

The term *pre-brief *refers to a constructive meeting between the learners and trainers held before or at the beginning of the simulation session. This pre-brief sets the scene and provides a forum for meaningful dialogue, allowing learners to express their expectations and resolve potential barriers to learning. It is also a formative learning tool as it helps in the identification of learning needs and learning gaps. The trainers should use the probe and enquiry technique to articulate the learners’ prior knowledge, identify learning gaps and formulate a mental plan on how to address these gaps during simulation. The trainers should set out the intended learning outcomes and agree on specific areas of interest the learners might want to focus on. This has been described to improve trainees' degree of participation and increase the collaboration between faculty members. The pre-brief also provides necessary information on the trainees’ learning styles and the most effective methods for delivering training to achieve learning outcomes [9].

During the Scenario

SOE provides an environment for learners to not only demonstrate that they *know* but also demonstrate the application of that knowledge [10]. This progression in learning can be demonstrated in the constructed clinical setting and observed as steady increases in performance levels by the trainer [11,12]. Formative assessment through simulation should involve conversations with the learner as this provides the opportunity to supervise this progress, fill knowledge gaps and ultimately transform the learner into a competent clinician for that task [6].

SOE provides a platform for curriculum integration and enhanced professional practice. It has been suggested that simulation if used appropriately could replace close to 25% of the curriculum [13,14]. It is a useful tool that could complement workplace assessment tools, which appraise observed behaviours of trainees such as mini-clinical evaluation exercises (mini-CEX) and direct observation of procedural skills (DOPS), which typically have low inter-rater reliability. The use of simulation in these cases may help in the standardisation of these procedural skills and may increase their reproducibility [13,14].

SOE can also be combined with clinical formative assessment tools, including mini-clinical examinations, case-based discussions and the DOPS [13]. Mini-CEX is a structured assessment designed to provide immediate feedback on clinical skills by observation of an actual clinical encounter. It has high reliability and feasibility and is generally acceptable. However, mini-CEX has drawbacks, including time constraints, high inter-rater variability and variations in scoring even among colleagues of the same grade [15,16]. Its reliability can be enhanced by having different trainers observe different encounters and applying it to a wide clinical content. It is an authentic form of assessment in near-natural settings compared with the conventional case presentation or objective structural clinical examination [17]. Case-based discussions explore trainees’ thought processes in clinical decision-making, their understanding of ethics as well as professional judgement. It is a reliable method of assessment, especially if sufficient numbers are done with many colleges advocating six to 12 assessments per year [18], and helps to consolidate learning achieved through simulation and direct observation such as mini-CEX.

Debrief

The debrief is a formative activity, which must involve the trainer who observed the learner's performance at a task to discuss their observations with the learner (concerning a standard) and engage in a discussion about their performance and ways it can be improved [19]. It is a learning conversation that provides individualised and tailored feedback on a learner’s performance [19]. A debrief must be a systematic re-examination of the learner's actions to reveal areas of strengths and weaknesses, directing further learning accordingly. The trainer should use *probing*, *enquiry* and *advocacy* techniques to understand the learners’ internal frames to comprehend the thought processes, assumptions or feelings that drive their actions [20]. This will help the trainers to engage in meaningful conversations advocating reasons why there should be a shift in the learners’ thought processing and culminates in refocusing on a proposed alternative with defined learning goals [20].

Effective Quality Assurance Processes

Quality assurance refers to activities aimed at sustaining good practices or improving current processes. To ensure that the aims of SOE, including improvement in knowledge, teamwork, communication and situation awareness, are met, there must be a process of objective evaluation of these objectives. These provide needed information for educational and clinical governance purposes and also help in the commitment of resources to meet educational needs.

## Review

Planning for the evaluation

It is important to gather the thoughts and opinions of the faculty of trainers, clinical staff and management staff in the design and delivery of an evaluation process or tool. This eliminates the resistance that could ensue in the acceptance of the findings of the evaluation and improves the process of dissemination and implementation of recommendations [21]. It also provides an opportunity to broaden the scope of evaluation to include aspects of the programme that could be relevant to the organisation.

A logic model is a useful tool in the planning, implementation and continuous evaluation of a programme. It provides a visual representation of the relationship between the resources available, programme activities and specified outcomes [22,23,24]. This model utilises flowcharts, diagrams and tables that itemise the various components of the programme, define and simplify the relationships between several steps, and enable critical analysis of each step involved in the delivery of the programme. In particular, this model is a useful framework and reference point in the assessment of the programme. It can be employed in the assessment of the feasibility of the programme at inception, clarifying goals and conceptual gaps, monitoring progress during implementation, and developing measures of evaluation and dissemination of knowledge [25]. The logic model is most beneficial if each component in the framework is linked to a quantifiable outcome [22,23,24].

Using the logic model [21] as a guide for the SOE programme, its programme activities would include the identification and preparation of the rooms in the labour ward, training the faculty of educators and the purchase of the mannequins. The *services delivered* include the training of the obstetric team (doctors, midwives and allied staff). It is anticipated that the cancellation of clinical activity and allocation of the study leave budget, which will ensure that staff members are released for this activity. The intermediate result (outcome indicator) of this programme is the assessment of the impact of SOE on the knowledge and behaviours of the attendees. The intended results (impact indicators) include an improvement in key obstetric performance indicators, including a reduction in the patient’s complaints and legal claims and an improvement in the patient experience.

The evaluation process

Evaluation of Knowledge Acquisition (Learning)

The main goal of the evaluation of SOE is the assessment of its impact on the learners’ acquisition of knowledge. In a review, Sawyer and Gray argued that simulation is a useful tool in the facilitation of competency evaluation and the maintenance of mastered skills. They described a well-designed simulation programme as a framework to acquire skills that can be transferred to real-life events [26]. Furthermore, Barsuk et al., in their review, were able to demonstrate the relevance of simulation-based learning (SBL) in facilitating skills retention and translate this to improvement in clinical care [27]. As such, simulation in the context of formative learning can be used in combination with other forms of workplace-based assessments such as case-based discussions, mini-clinical examinations and direct observation of procedural skills to facilitate and promote learning.

To objectively assess the impact that the programme has on improving learning, that is, if the learners improved their knowledge base or acquired new knowledge, it is necessary to define their knowledge base before attending the course (simulation). This can be achieved through a pre-simulation assessment test using a well-designed self-reported questionnaire. The utilisation of a self-reported questionnaire survey has the advantages of being economical, cost-beneficial and generating quick responses. Dillman et al. argued further that the response rates may be higher if completed as a self-reported questionnaire [28]. Moreover, the utilisation of the internet for survey delivery ensures that it is accessible and convenient while also reducing the cost of administration [28,29]. It also allows for quick and direct responses from participants and eliminates *content* transfer errors. The internet facilitates the use of available software in data analysis [28,29] and encourages the completion of the survey in privacy, which promotes authentic responses from the participants [28]. From a qualitative assurance perspective, it is pertinent that the assessment tool be acceptable, reliable, valid and feasible and of educational value [30]. The content of the questionnaire should be validated by all faculty members and invited experts in the field. Krosnick and Presser reiterated the importance of colleague feedback in the development of a tool for evaluation, particularly in the validation of the contents for their readability, understanding of the content (items) of the questionnaire and practicality of the evaluation [28,31]. It also ensures that the items are applicable and relevant to the overall intentions of the programme [28,31]. The survey questions should be itemised clearly and presented in a simple and comprehensible format. Survey as a method of assessment has a reliable test/retest reliability in the measurement of learning. The responses from the same individual make them reliable, authentic and reproducible [30].

The evaluation of SOE should involve a pre-test (written or verbal assessment of prior knowledge), which is ideal and useful in identifying the knowledge gaps to plan and direct learning [7]. It also provides a basis for comparison, particularly when compared to subsequent assessments in formative learning. The pre-test questionnaire items should contain all aspects of obstetric simulation to be covered within the course and include details such as professional background and level of experience. The post-test assessment (survey) should be based on the same set of questions and collected after the course. The completion of the survey afterwards provides a true reflection of the effect and value of the programme in improving learning, and completion rates are improved if it is a prerequisite to the collection of attendance certificates. Some schools of thought suggest that there should be a time lag following the completion of the course to allow participants to reflect on the skills acquired and determine the educational value of the programme. However, in practice, there is an association between decreased compliance and recall bias that may affect the response rates and accuracy of responses. A comparative analysis of the pre- and post-simulation tests would identify knowledge gaps and areas for improvement. The differences in the scores of the pre- and post-tests correlate to additional knowledge acquired on the programme and would reflect the value of the programme in improving clinical knowledge.

Evaluation of Processes Involved in Service Provision

SOE provides an opportunity to examine the processes involved in the provision of clinical care during emergencies. It provides a microscopic examination of the logistics and support services available in obstetric emergencies such as timely access to blood services in emergencies, etc. It provides the opportunity for critical examination of these processes to identify where lapses in the system are and how these can be managed.

The assessment should begin with the itemisation of all processes involved in each simulated obstetric emergency. This enables visibility of these processes to identify areas of bottleneck and non-value-adding steps. This will enable the managers to eliminate such steps and streamline response times during emergencies. The revised steps should be simulated in the future, and a continuous process of evaluation can be integrated and imbibed into the culture of the organisation.

This can be achieved by interviewing learners and trainers following the SOE and gathering information on their experiences and how they feel the processes can be enhanced. It is important to identify common themes, which can be gathered by identifying the unifying concepts, perceptions and lessons learnt [32]. These themes should be addressed by management and conclusions compared with the literature to generate new policies that can improve local practice.

Evaluation of the Impact of SOE

The value and impact of the programme should be assessed and should entail an analysis of the cost incurred in setting up and implementing SOE compared with the outcome indicators [21]. Costs should not only include direct expenses, such as the purchase of mannequins but should also take into account indirect costs, particularly opportunity costs, such as the cancellations of clinics, the cost of study leave for participants and time off in lieu for the trainers. This would inform the costs per trainee and may influence decisions about the management of the programme such as the timing of SBL and how best to integrate with minimal disruption to other departmental activities. It would also provide information on resource allocation and budgetary allowance for the programme [21]. A cost-effectiveness analysis is conducted to understand if the programme meets its objectives at a reasonable and sustainable budget and can be achieved by analyzing data on the impact indicators, such as the number of adverse outcomes, the number of department patient complaints received, the costs of legal claims and budgetary allocations for contribution to the clinical negligence scheme by the health board for maternity services [33]. Successful programmes should see a positive trend in these figures, with a reduction in the number or severity of adverse incidents, the number of patient complaints and contributions to the negligence scheme. These analyses may have cost implications but are useful to understand how best to fund the programme and prioritisation and appropriate allocation of scarce resources to improve clinical service delivery to meet clinical needs and outcomes. Moreover, information on patient experience must be gathered through the collection of a patient satisfaction survey following live obstetric emergencies and assessed to understand if current processes are adequate. Furthermore, there should be a quantitative and qualitative annual review of complied complaints or compliments to look for trends that require improvement that can be incorporated into subsequent SOE.

Evaluation of Trainers

SOE is useful to provide information to the management team on the current processes guiding educational delivery and may unearth the absence of necessary skills or gaps in the expertise required for the delivery of certain aspects of training. Therefore, trainers must be assessed for feedback preferably through the use of a questionnaire survey as it is quick, cheap and easy to collate. This should be combined with interviews that understand the contexts of issues identified in the responses collated through the survey. Furthermore, it is recommended that annual or biannual update courses are provided for the faculty of trainers to keep abreast of new technologies and advancements in patient care. This provides an opportunity for continuous professional development for these trainers and also an additional level of quality assurance for clinical practices. These professional development opportunity sessions may attract financial investments but have been shown to have positive impacts such as improving the quality of feedback provided to trainees by trainers and improving the trainers’ teaching techniques that have increased trainee satisfaction rates SBL [17].

Dissemination of the Evaluation Report

The evaluation report should not only contain findings but also analyse and synthesise them to conclude the programme. It is also necessary to reflect on these conclusions within the context of the programme to gain a clear understanding of identified issues and how they can be addressed [21].

The conclusions of the report must be disseminated in an efficient and timely manner to all stakeholders. This would promote stakeholder engagement and would facilitate the adoption of recommendations within the service. In this case, the findings should be presented at the next available obstetric governance meetings to all shareholders, including obstetricians, midwives and managers. This provides a platform for discussion and justification of the findings of the evaluation and enables productive discussions on how to improve the programme and safe practices within local obstetric units. The recommendations should be communicated formally in a timely and unbiased manner to all members of staff and should be referenced in their professional development portfolio. The implementation of the recommendations may necessitate programme redesign and the creation of policies, and it should be facilitated by the managers. These should be in line with SMART principles, that is, the policies should be specific, measurable, attainable, realistic and timely. This enables continuous assessment of the impact of these policies and provides opportunities to further improve clinical practices and, ultimately, patient outcomes.

What Is the Best Way to Deliver SOE?

An in situ model of learning is one where learning takes place in the actual work environment or clinical facility, involving those who deliver the care [1]. This differs from an off-site or centre-based model where learning occurs in a different location or facility. Recently, it has been suggested that an in situ model of SBL for obstetric emergencies may be more effective than an off-site model [1]. In their review, Sørensen et al. supported the view that training within the natural environment where actual clinical care will be delivered has the advantage of identifying potential obstacles in the labour ward [1].

The delivery of SBL as an in situ model ensures that learners find the application of the knowledge and skills acquired more relevant and applicable in the local context, particularly regarding the available skill mix and resources [1,3]. It also has the advantage of psychological fidelity and may be used as part of assessment processes in the introduction of new procedural techniques in the labour ward. It also has the potential benefits of cost savings compared to the off-site SBL, which incurs the costs of securing facilities and transportation, to mention a few; and trainers are also able to relate to local problems and offer solutions that can improve healthcare delivery.

However, the in situ model of SBL has the drawback of familiarity amongst colleagues, which could affect how seriously members of the team engage with the session. This shortcoming can be mitigated by ensuring that resources, such as staff budgetary allowances, are allocated to these training sessions and clear time slots are guaranteed for each learner. This system makes it necessary to attend these sessions because team members understand that part of their study leave budget goes towards these mandatory learning events. It should also be included as part of their requirements for a successful annual job appraisal.

## Conclusions

Simulation is an ideal method of procedural training in obstetrics. To maximise training opportunities during simulation, the evaluation of these educational activities should be based on a standardised, evidence-based approach. The tools used in the evaluative process should be validated for content and context. This ensures good quality assurance and consistency of approach and makes the findings and recommendations derived credible, acceptable and applicable. Moreover, the information derived from the evaluation can be used for planning further learning, assessing the competency of the faculty and for educational governance purposes. In our view, simulation should be used in conjunction with other forms of procedural assessment such as mini-clinical examinations and case-based discussions in the translation of these skills into actual life events. Learners are then able to further consolidate their learning, improve their professional skills and feel involved throughout the programme.

In addition to improving a learner’s knowledge base, SOE helps facilitate and assess interprofessional and collaborative learning by strengthening team bonding and improving communication, thereby reducing adverse incidents that may result from *human factors*. It also enables the learners to understand their roles and appreciate how these fit with the overall patient care.

## References

[1] Sørensen JL, Van der Vleuten C, Lindschou J (2013). “In situ simulation” versus “off site simulation” in obstetric emergencies and their effect on knowledge, safety attitudes, team performance, stress, and motivation: study protocol for a randomized controlled trial. Trials.

[2] Merién AE, van de Ven J, Mol BW, Houterman S, Oei SG (2010). Multidisciplinary team training in a simulation setting for acute obstetric emergencies: a systematic review. Obstet Gynecol.

[3] Crofts JF, Ellis D, Draycott TJ, Winter C, Hunt LP, Akande VA (2007). Change in knowledge of midwives and obstetricians following obstetric emergency training: a randomised controlled trial of local hospital, simulation centre and teamwork training. BJOG.

[4] Ellis D, Crofts JF, Hunt LP, Read M, Fox R, James M (2008). Hospital, simulation center, and teamwork training for eclampsia management: a randomized controlled trial. Obstet Gynecol.

[5] Kumar A, Sturrock S, Wallace EM (2018). Evaluation of learning from practical obstetric multi-professional training and its impact on patient outcomes in Australia using Kirkpatrick’s framework: a mixed methods study. BMJ Open.

[6] Khan K, Ramachandran S (2012). Conceptual framework for performance assessment: competency, competence and performance in the context of assessments in healthcare--deciphering the terminology. Med Teach.

[7] Taylor DC, Hamdy H (2013). Adult learning theories: implications for learning and teaching in medical education: AMEE Guide No. 83. Med Teach.

[8] Knowles MS, Holton EF III, Swanson RA, Robinson PA (2020). The Adult Learner: The Definitive Classic in Adult Education and Human Resource Development.

[9] Wertsch JV, Sohmer R (1995). Vygotsky on learning and development. Hum Dev.

[10] Miller GE (1990). The assessment of clinical skills/competence/performance. Acad Med.

[11] Issenberg SB, McGaghie WC, Petrusa ER, Lee Gordon D, Scalese RJ (2005). Features and uses of high-fidelity medical simulations that lead to effective learning: a BEME systematic review. Med Teach.

[12] Bradley P (2006). The history of simulation in medical education and possible future directions. Med Educ.

[13] Hayden JK, Smiley RA, Alexander M (2014). The NCSBN National Simulation Study: a longitudinal, randomized, controlled study replacing clinical hours with simulation in prelicensure nursing education. J Nurs Regul.

[14] Watson K, Wright A, Morris N (2012). Can simulation replace part of clinical time? Two parallel randomised controlled trials. Med Educ.

[15] Boursicot K, Etheridge L, Setna Z, Sturrock A, Ker J, Smee S, Sambandam E (2011). Performance in assessment: consensus statement and recommendations from the Ottawa conference. Med Teach.

[16] Kogan JR, Bellini LM, Shea JA (2003). Feasibility, reliability, and validity of the mini-clinical evaluation exercise (mCEX) in a medicine core clerkship. Acad Med.

[17] Singh T, Sharma M (2010). Mini-clinical examination (CEX) as a tool for formative assessment. Natl Med J India.

[18] Etheridge L, Boursicot K (2017). Performance and Workplace Assessment. A Practical Guide for Medical Teachers, 6th edn.

[19] Tavares W, Eppich W, Cheng A, Miller S, Teunissen PW, Watling CJ, Sargeant J (2020). Learning conversations: an analysis of the theoretical roots and their manifestations of feedback and debriefing in medical education. Acad Med.

[20] Rudolph JW, Simon R, Dufresne RL, Raemer DB (2006). There's no such thing as "nonjudgmental" debriefing: a theory and method for debriefing with good judgment. Simul Healthc.

[21] Centers for Disease Control and Prevention (2001). Program Operations Guidelines for STD Prevention. US Department of Health and Human Services, pp.S1-S39.

[22] Scott SJ, Denne LD, Hastings RP (2018). Developing a logic model to guide evaluation of impact for learning disability projects: the case of the Positive Behavioural Support (PBS) Academy. Tizard Learn Disabil Rev.

[23] Kaplan SA, Garrett KE (2005). The use of logic models by community-based initiatives. Eval Program Plann.

[24] Renger R, Foltysova J, Becker KL, Souvannasacd E (2015). The power of the context map: designing realistic outcome evaluation strategies and other unanticipated benefits. Eval Program Plann.

[25] Ebenso B, Manzano A, Uzochukwu B (2019). Dealing with context in logic model development: reflections from a realist evaluation of a community health worker programme in Nigeria. Eval Program Plann.

[26] Sawyer T, Gray MM (2016). Procedural training and assessment of competency utilizing simulation. Semin Perinatol.

[27] Barsuk JH, Cohen ER, Williams MV (2018). Simulation-based mastery learning for thoracentesis skills improves patient outcomes: a randomized trial. Acad Med.

[28] Dillman DA, Smyth JD, Christian LM (2014). Internet, Phone, Mail, and Mixed Mode Surveys: The Tailored Design Method, 4th edn.

[29] Roberts LD, Allen PJ (2015). Exploring ethical issues associated with using online surveys in educational research. Educ Res Eval.

[30] Huwendiek S, De Leng BA, Kononowicz AA (2015). Exploring the validity and reliability of a questionnaire for evaluating virtual patient design with a special emphasis on fostering clinical reasoning. Med Teach.

[31] Tavakol M, Sandars J (2014). Quantitative and qualitative methods in medical education research: AMEE Guide No 90: Part I. Med Teach.

[32] Krosnick JA, Presser S (2009). Question and Questionnaire Design. Stanford University.

[33] Haddix MM (2014). Professional resources. J Adolesc Adult Lit.

